# Validation Study: Response-Predictive Gene Expression Profiling of Glioma Progenitor Cells *In Vitro*

**DOI:** 10.1371/journal.pone.0151312

**Published:** 2016-03-15

**Authors:** Sylvia Moeckel, Arabel Vollmann-Zwerenz, Martin Proescholdt, Alexander Brawanski, Markus J. Riemenschneider, Ulrich Bogdahn, Anja-Katrin Bosserhoff, Rainer Spang, Peter Hau

**Affiliations:** 1 Department of Neurology and Wilhelm Sander-NeuroOncology Unit, University Hospital Regensburg, Regensburg, Germany; 2 Department of Neurosurgery, University Hospital Regensburg, Regensburg, Germany; 3 Department of Neuropathology, Regensburg University Hospital, Regensburg, Germany; 4 Institute of Biochemistry (Emil Fischer-Zentrum), Friedrich-Alexander Universität Erlangen-Nürnberg, Erlangen, Germany; 5 Institute for Functional Genomics, University of Regensburg, Regensburg, Germany; Medical University Graz, AUSTRIA

## Abstract

**Background:**

In a previous publication we introduced a novel approach to identify genes that hold predictive information about treatment outcome. A linear regression model was fitted by using the least angle regression algorithm (LARS) with the expression profiles of a construction set of 18 glioma progenitor cells enhanced for brain tumor initiating cells (BTIC) before and after *in vitro* treatment with the tyrosine kinase inhibitor Sunitinib. Profiles from treated progenitor cells allowed predicting therapy-induced impairment of proliferation *in vitro*. Prediction performance was validated in leave one out cross validation.

**Methods:**

In this study, we used an additional validation set of 18 serum-free short-term treated *in vitro* cell cultures to test the predictive properties of the signature in an independent cohort. We assessed proliferation rates together with transcriptome-wide expression profiles after Sunitinib treatment of each individual cell culture, following the methods of the previous publication.

**Results:**

We confirmed treatment-induced expression changes in our validation set, but our signature failed to predict proliferation inhibition. Neither re-calculation of the combined dataset with all 36 BTIC cultures nor separation of samples into TCGA subclasses did generate a proliferation prediction.

**Conclusion:**

Although the gene signature published from our construction set exhibited good prediction accuracy in cross validation, we were not able to validate the signature in an independent validation data set. Reasons could be regression to the mean, the moderate numbers of samples, or too low differences in the response to proliferation inhibition in the validation set. At this stage and based on the presented results, we conclude that the signature does not warrant further developmental steps towards clinical application.

## Introduction

The clinical management of gliomas, especially glioblastoma (GBM), is challenging, and outcomes are poor with a median survival time of only 14.6 months after standard radio-chemotherapy [1]. Novel treatment approaches are therefore urgently warranted. Treatment decisions for GBM patients are based on clinical factors and molecular markers like *MGMT* promoter methylation [2]. Recent genomic studies established sub-classifications of GBMs based on gene expression profiling [3, 4] or integrated genetic and epigenetic profiling [5]. These GBM subtypes were associated with distinct prognosis, however, no specific treatment selection including novel targeted agents can be derived from these classifications.

Recently, we presented a novel approach to identify genes that hold predictive information about treatment outcome [6]. Sunitinib was used as a model substance as it generally failed within clinical trials in gliomas [7–11], but generated responses in small subsets of patients. We used 18 short-term serum-free cultures of high-grade gliomas enhanced for brain tumor initiating cells (BTIC) before and after *in vitro* treatment with the tyrosine kinase inhibitor Sunitinib to predict treatment response *in vitro*.

Gene expression profiles from treated progenitor cells allowed predicting therapy-induced impairment of proliferation in vitro. In particular, we applied *least angle regression* [12] to simultaneously identify a set of signature genes, the optimal number of signature genes, and weights for the chosen genes. Proliferation 96 hours after treatment was predicted using the resulting weighted average of expression of the identified genes. Predictions were done in leave one out cross validation. The correlation between predicted and observed proliferation 96 hours after treatment were significant (p = 0.003).

We assumed that the selected signature genes revealed important information that could be used in the context of patient treatment if it was possible to demonstrate a good predictive quality in an independent data set. We wondered if clinical responses could be predicted in an *in vitro* setting, and if this knowledge could be translated in a clinical setting. The study presented here was conducted with 18 additional BTIC cultures to validate the predictive properties of the signature.

## Materials and Methods

### Tumor samples and patient characteristics

Native glioma tissue samples were obtained from 18 patients undergoing surgical resection at the local Department of Neurosurgery with a diagnosis of high-grade glioma WHO grade III or IV. All tumors were histologically classified according to the 2007 WHO classification of tumors of the central nervous system by the local neuropathologist (MJR). Specimens were cultured according to current criteria for the culture of brain tumor initiating cells (BTIC) [13]. All patients gave written informed consent, and this study and further use of the samples were specifically approved by the ethics committee of the University of Regensburg, Regensburg, Germany (No° 11-103-0182).

### Primary cell culture of brain tumor initiating cells (BTICs)

Tissue samples were kept in PBS at 4°C and processed within 24 hours after surgery. Further procedures are described in the primary publication [6]. The lowest available passage of all BTIC primary cultures (usually below passage 8) was used for all assays.

### Treatment of BTIC cultures with Sunitinib

Sunitinib was purchased from Sigma Aldrich (St. Louis, Missouri, USA) and prepared as a 25 mmol/l stock solution in DMSO for *in vitro* studies. BTICs were grown in cell culture dishes (TPP, Trasadingen, Switzerland) until they formed a subconfluent monolayer (density of 80%). Laminin coated dishes were used for cells that grew non-adherent under neurosphere conditions. Before treatment, cells were cultured in growth factor free medium for 16 hours to simulate *in vivo* conditions. After starvation, cells were treated with 1 μM Sunitinib in the treatment groups or 0.00025% DMSO in the control groups with supplementation of recombinant growth factors PDGF-A/B and VEGFA (25 ng/ml) for 6 hours before harvest. Cells were either harvested in RLT-lysis buffer (provided in the RNeasy Kit, Qiagen, Hilden, Germany) for subsequent RNA-extraction. For Western Blot analysis, cells were lysed in RIPA buffer (50 mM Tris, 150 mM NaCl, 0.5% Triton X100, 0.5% Deoxycholate, 4-(2-Aminoethyl)benzenesulfonyl fluoride hydrochloride, Halt™ Protease Inhibitor Cocktail; Thermo Scientific, Massachusetts, USA).

### Microarray analysis

Hybridization to arrays was performed in the local Competence Center for Fluorescent Bioanalytics. Quality of RNA was confirmed by HPLC, and RNA was further processed by reverse transcription. cDNA was converted to Biotin-labeled cRNA which was then hybridized to Affymetrix hugene.1.1.st GeneChips (Affymetrix, Santa Clara, California, USA).

Microarray data are deposited at the gene expression omnibus (GEO) functional genomics data repository under accession number GSE76990.

### Western blot analysis

For Western blot analysis, 15 μg of total cell lysates were used. Detailed procedures are described elsewhere [6].

### Proliferation assay

BTICs were treated with 1 μM Sunitinib in the treatment group or the corresponding DMSO concentration in the control group in 5 replicates. Cellular viability was assessed by the Cell Proliferation Kit II (XTT) from Roche Applied Science (Roche, Basel, Germany) according to the manufactures protocol. Photometric evaluation was performed with a Varioscan ELISA reader (Thermo Scientific, Massachusetts, USA). Relative absorption of treated cells compared to untreated control was defined as proliferation rate. For every individual BTIC line, the XTT assay was repeated at least three times.

### Computational Analysis and Statistics

Computational analysis was performed using R and Bionconductor (http://bioconductor.org). Normalization and pre-processing methods were performed as previously described. For prediction of proliferation rates, we used the multiple regression model which has been introduced previously [6].

If not specified otherwise statistical analysis of *in vitro* data was performed using the student’s t-test. A p-value less than 0,05 was considered to be statistically significant (*, p < 0,05; ** p < 0,01, *** p< 0,001).

## Results

### Characterization of patient material

**Fig 1:**
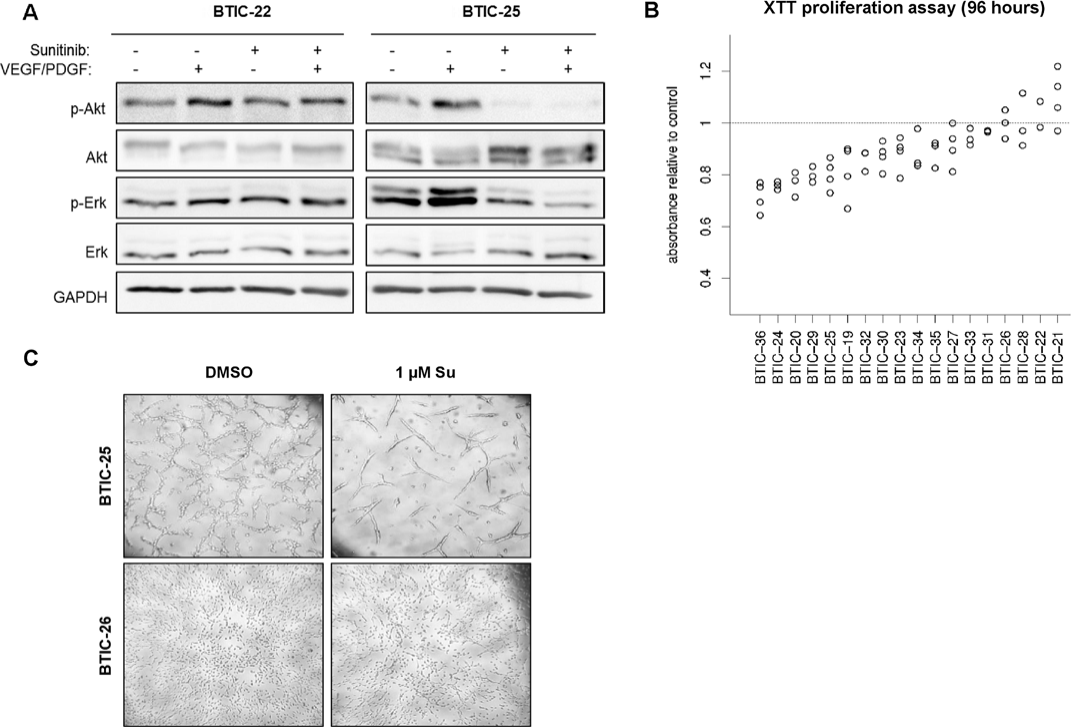
Proliferation and pathway-modulation by Sunitinib. (A) Western Blot analysis to analyze AKT phosphorylation (Ser473) was performed with 18 BTIC lines of which 2 representatives are shown. To evaluate distinct phosphorylation patterns under treatment, BTIC were treated with 1 μM Sunitinib or 0.00025% DMSO for 6 hours with growth factor supplementation (25 ng/ml) as outlined. GAPDH was used as protein loading control. (B) BTICs were incubated with 1 μM Sunitinib or 0.00025% DMSO (control), and the XTT proliferation assay was performed after 96 h. The relative difference of the mean proliferation relative to control is blotted in a dot blot graph (y-axis) against the corresponding BTIC line (x-axis). Each data point indicates the result of an individual experiment. (C) Growth pattern in a responding (BTIC-25) and a non-responding (BTIC-26) BTIC line. Representative pictures are shown for two differently responding BTIC lines.

Eighteen native glioma tissue samples were obtained from patients undergoing surgical resection at the local Department of Neurosurgery. Tumors were neuropathologically classified as GBM in all 18 cases. Patient characteristics and genetic markers (IDH-1 mutation, MGMT promoter methylation and the subtype genetic signature of the respective tumor samples) were assessed as described before [6] (Table 1). The median age at diagnosis of our patient cohort was 65 years, and the median survival of 14 months (Table 1) was in the range of published data. The percentage of tumors with methylated MGMT-Promotor (4/17 = 24%), which is considered as an important prognostic marker in high-grade gliomas, corresponds with the finding of other studies [4]. In line with our initial study, we were able to determine two distinct molecular subtypes (mesenchymal and proneural, respectively) from the initial patient material. The primary cell cultures (BTICs) displayed highly individual morphologies and *in vitro* growth patterns (Table 1, Fig 1C, data not shown).

**Table 1:** Clinical and biological information of parental tumor specimen from the analyzed BTIC panel vs. BTIC lines.

BTIC	Original tumor				Patient characteristics			Primary cell culture	
	Histology	WHO-Grade (I-IV)	MGMT-Methyl.	IDH1 (wt/mut.)	Age	Gender	Survival (/months)	*in vitro* growth	Molecular Classification
**19**	GBM (prim.)	IV	unmeth.	wt	71	m	10	spheres	proneural
**20**	GBM (prim.)	IV	unmeth.	wt	63	m	16	spheres	proneural
**21**	GBM (prim.)	IV	unmeth.	n.d.	71	m	23	spheres	proneural
**22**	GBM (prim.)	IV	meth.	wt	72	m	21	spheres	proneural
**23**	GBM (prim.)	IV	n.d.	wt	69	m	8	semi-adherent	mesenchymal
**24**	GBM (prim.)	IV	unmeth.	wt	74	f	14	spheres	proneural
**25**	GBM (prim.)	IV	meth.	wt	41	m	#	adherent	proneural
**26**	GBM (prim.)	IV	unmeth.	wt	59	m	8	adherent	mesenchymal
**27**	GBM (prim.)	IV	unmeth.	wt	61	f	30	semi-adherent	mesenchymal
**28**	GBM (prim.)	IV	unmeth.	wt	68	m	4	spheres	proneural
**29**	GBM (prim.)	IV	unmeth.	wt	75	f	8	adherent	mesenchymal
**30**	GBM (prim.)	IV	unmeth.	wt	64	m	16	spheres	proneural
**31**	GBM (prim.)	IV	unmeth.	wt	67	m	5	spheres	proneural
**32**	GBM (prim.)	IV	unmeth.	wt	59	f	#	semi-adherent	proneural
**33**	GBM (prim.)	IV	meth.	wt	59	f	#	semi-adherent	proneural
**34**	GBM (prim.)	IV	meth.	wt	58	m	13	adherent	proneural
**35**	GBM (prim.)	IV	unmeth.	wt	61	f	21	semi-adherent	proneural
**36**	GBM (prim.)	IV	unmeth.	wt	86	m	10	adherent	mesenchymal

Histology and WHO tumor grade were evaluated in the original tumors by an independent neuropathologist (MR). MGMT = Methyl-Guanine-Methyl-Transferase; meth. = methylated MGMT-Promotor (> 8%); unmeth. = unmethylated MGMT-Promotor; IDH = isocitrate dehydrogenase; wt = wild type; n.d. = not determined; f = female; m = male; # = censored cases; all patients underwent first-line therapy with radiotherapy of 60 Gy plus concomitant chemotherapy (Temozolomide 75 mg/m2 daily during radiotherapy), then adjuvant Temozolomide 150–200 mg/m2 d1-5 in 28 days until progression or for up to 6 cycles.

In summary, the general characterization of our validation dataset was in good agreement with the construction dataset. Furthermore, based on the clinical and demographical data, we could exclude that the *in vitro* conditions select for a specific phenotype.

### The validation dataset confirms heterogeneity of treatment response on a molecular and functional level

In our preceding work, we observed heterogeneous responses to Sunitinib treatment on the level of pathway activation, signal propagation, and target protein expression [6].

Growth factor supplementation was used to examine the inhibition of pre-stimulated pathways. Growth factor free conditions were chosen to investigate the blockage of autocrine and paracrine pathway activation. Nevertheless, here and previously, semi-quantitative Western blot analysis of a selected panel of transmitter molecules did not provide sufficient information for the prediction of treatment effects. Therefore, we did not investigate the pathway activity in the validation cohort. However, AKT and ERK activity were analyzed in 2 representative BTIC cultures to confirm the heterogeneous nature of pathway activation and inhibition (Fig 1A).

In the earlier study, we developed a gene signature predicting proliferation 96 hours after treatment [6]. In line with our previous results, we here observed variable responses (Fig 1B and 1C). Sunitinib affected the number of viable cells in 14 BTICs (78%), which was significant in 10 BTIC cultures (56%, p<0.05). A small set of 4 BTIC lines exhibited no detectable response at all. However, a maximum inhibition of proliferation of up to 35% was reached compared to 56% in our previous study, indicating a smaller range of treatment responses in the validation dataset.

### Genome wide expression profiles of the construction and validation dataset are devoid of a batch specific bias

Transcriptome-wide expression profiles of BTIC enriched cell cultures were generated before and 6 hours after *in vitro* treatment with Sunitinib using Affymetrix hugene.1.1.st GeneChips.

All 18 BTIC lines were treated with 1 μM Sunitinib or 0.00025% DMSO with supplementation of VEGF and PDGF-AB for 6 hours after overnight starvation in serum- and growth factor-free medium.

**Fig 2:**
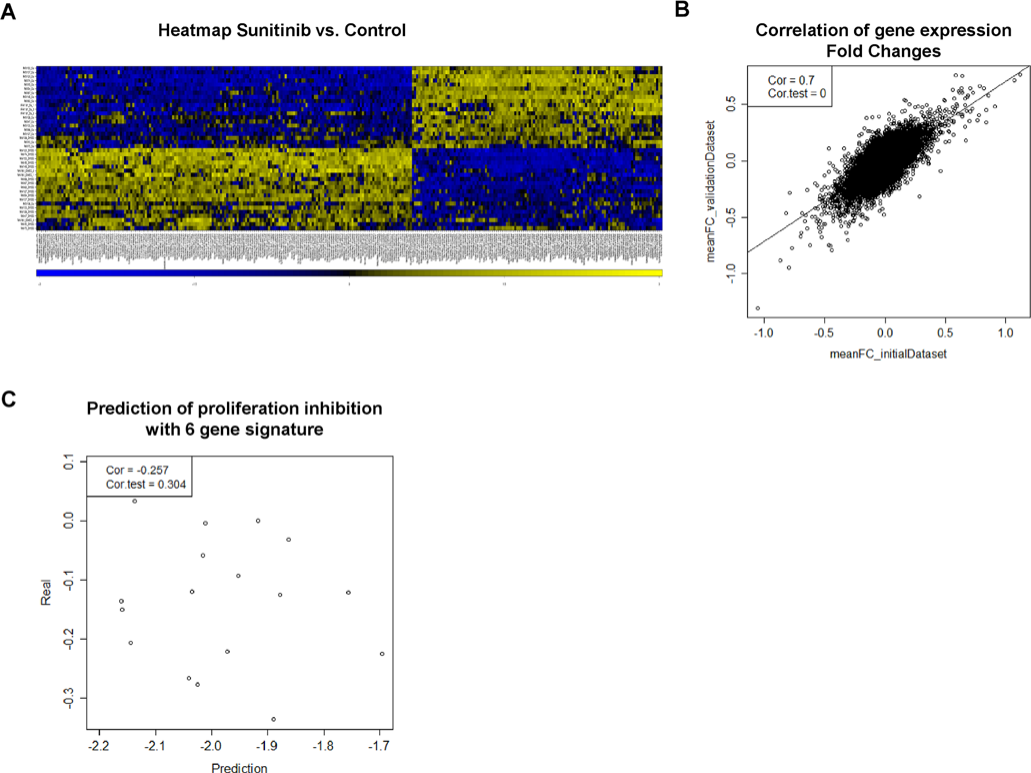
Gene expression pattern and prediction of proliferation. (A) Heat map of the 300 most differentially expressed genes when comparing Sunitinib treated with untreated samples. The samples are nicely separated into treated vs. untreated samples. (B) The FC (fold change) expression difference between DMSO and Sunitinib treated samples were calculated for each gene with expression values obtained from construction and validation data sets, respectively. The correlation of FC values is shown as a scatterplot (correlation coefficient, 0.7; p<0.001). (C) Proliferation inhibition after 96 hours treatment was predicted by calculating the sum of weighted expression of 6 signature genes (CLK4, BCLAF1, LOC100130581, ACTG, VAV3, DPF3). Predicted proliferation inhibition was plotted against the average relative proliferation inhibition (see Fig 1B) (correlation coefficient, -0.257; p = 0.304).

All data analysis was restricted to the 500 genes with the highest expression variances across all samples from the validation dataset. As seen in the prior study, the expression differences between patients were larger than differences before and after treatment (S1A Fig). Again, we applied the batch effect correction algorithm Combat in order to zoom in on treatment effects which resulted in a clear separation of treated and untreated samples (Fig 2A and S1B Fig). Interestingly, hierarchical clustering of the combined microarray data from both cohorts did not separate the samples in the construction and validation data sets (S1C Fig) which, to a certain extent, dispel the concern of a technical bias in data collection. To further elucidate the inter-experimental bias, we computed the fold change (FC) expression between control and Sunitinib treated samples (both supplemented with VEGF/PDGF) of each gene. FC expression of the initial dataset was plotted against the corresponding FC expression in the validation dataset (Fig 2B). We observed a high correlation (r = 0.7; p<0.001; Pearson correlation) between both datasets, showing good reproducibility of the treatment effect on gene expression.

### The predefined gene expression signature does not exhibit predictive power

In order to validate the signature proposed in Moeckel et al. [6], we calculated the response rate based on the multiple regression model and the expression values of the signature genes of Sunitinib-treated samples retrieved from the validation dataset. The lack of a correlation between the actual measured proliferation impairment (see Fig 1B) and predicted (calculated) proliferation inhibition (Fig 2C) indicates that the gene signature is not predictive in an independent experimental setup.

Separation of samples into TCGA subclasses does not revoke the lack of prediction accuracy (S2A Fig). Furthermore, we did not derive a new prediction signature when we applied the *Least angle regression* algorithm to the combined dataset with all 36 BTIC cultures (S2B Fig).

## Discussion

In a previous publication, we introduced a novel approach to identify genes that hold predictive information about treatment outcome. In a validation set of 18 BTIC primary cultures presented here, we were not able to validate the microarray-based response prediction signature [6]. Based on the presented results, we conclude that the signature does not warrant further developmental steps towards clinical application at this time.

There are multiple explanations for our failure to reproduce the predictivity of our signature. The most important one may be a conceptual difference between statistical significance and reproducibility. In spite of the good correlation of 0.7 for the reproduction of treatment effects across genes, there is regression to the mean: The strongest expression differences in the first study were reduced in the second. This is a normal phenomenon in every reproducibility study. It becomes stronger if the estimation errors of expression differences are higher. These errors depend on the size of the study. Indeed, one major constraint of our approach is the limitation of sample size, most importantly of our training set, which was restricted to 18 BTIC cultures. In our case, regression to the mean might have been sufficient to dissolve the signature's predictability, highlighting the importance of reproducibility studies in general.

Although we strictly followed the protocols from our prior study and ruled out the possibility of a systemic batch specific bias, our test sets might be still biased for factors that were not controlled for, which would lead to distinct response patterns in both datasets that only included moderate numbers of samples. In fact, experimental details of stable *in vitro* expansion procedures that influence the dynamic phenotypic plasticity of cancer stem cells have not been fully elucidated yet. Taking into account the complexity of incoming signals in an *in vivo* setting, slight fluctuation of the *in vitro* culture environment should not be crucial.

Another important aspect is the small variability of the response variable (here proliferation inhibition), especially in the validation set. Indeed, we measured proliferation inhibition of up to 56% in several BTIC lines in our training set, whereas inhibition did not exceed 35% in the validation set presented here. We refused to increase the Sunitinib dosage as it would not represent any reachable physiologic condition. Nevertheless, our results may indicate that these small differences cannot be described by gene transcription levels, and that the smaller differences in the validation set might have diluted the results of the full cohort if the sets were analyzed in combination.

In spite of the failure of our signature, *in vitro* drug testing tools for predicting treatment effects in tumor patients are urgently needed to prevent overtreatment of patients who are not susceptible to response and to avoid negative clinical trials. Based on our now negative results, the necessity of early prediction of response, the experience that only a small subset of patients responds in clinical trials using small molecule inhibitors as Sunitinib [7–11], and the urgent medical need, we suggest to further develop the field of *in vitro* drug testing, despite decades of failures. We argue that it is important to experimentally ask the right cells the right questions. Progenitor cells selected by short-term *in vitro* culture and treated over short periods with an agent in question before evaluation of response may still provide a promising tool. However, the molecular correlate of such an assay is still not obvious. Our preclinical study shows that our strategy yielded evaluable results that might be developed to a more sophisticated assay. Such an approach could be useful in clinical trials and in the practice setting alike.

## Supporting Information

S1 Fig Correction for BTIC line specific expression variances enables the detection of treatment specific expression variances. (A) The 500 most variable genes of the construction data set were hierarchically clustered according to Euclidean distances showing that treatment and control (DMSO) samples cluster within the corresponding BTIC line. (B) After compensation for inter-tumoral variability using the batch effect correction algorithm Combat, samples clustered mainly within treatment specific groups. (C) The hierarchical cluster of the 500 most variable genes of the combined dataset shows no separation of construction and validation samples.
(TIFF)

S2 Fig Subclass-specific proliferation prediction and prediction with a whole-dataset derived signature. (A) Proliferation inhibition after 96 hours of treatment was predicted by calculating the sum of weighted expression of 6 signature genes (CLK4, BCLAF1, LOC100130581, ACTG, VAV3, DPF3). Predicted proliferation inhibition was plotted against the average relative proliferation inhibition (see Fig 2C). Data points corresponding to proneural BTICs were labeled blue and mesenchymal BTIC were labeled red, respectively. The Pearson’s correlation coefficients were calculated for each subclass separately and are shown in the upper-left plot legend. (B) Predicted proliferation inhibition was plotted against measured proliferation rates after treatment for all 36 BTIC cultures after running model selection with 36 samples as a training set.
(TIFF)

## References

[1] Stupp R, Mason WP, van den Bent MJ, Weller M, Fisher B, Taphoorn MJ, et al Radiotherapy plus concomitant and adjuvant temozolomide for glioblastoma. N Engl J Med. 2005;352(10):987–96. . 15758009 10.1056/NEJMoa043330

[2] Weller M, Stupp R, Hegi ME, van den Bent M, Tonn JC, Sanson M, et al Personalized care in neuro-oncology coming of age: why we need MGMT and 1p/19q testing for malignant glioma patients in clinical practice. Neuro-oncology. 2012;14 Suppl 4:iv100–iv8. Epub 2012/11/01. 10.1093/neuonc/nos206 23095825 PMC3480248

[3] Phillips HS, Kharbanda S, Chen R, Forrest WF, Soriano RH, Wu TD, et al Molecular subclasses of high-grade glioma predict prognosis, delineate a pattern of disease progression, and resemble stages in neurogenesis. Cancer Cell. 2006;9(3):157–73. . 16530701 10.1016/j.ccr.2006.02.019

[4] Verhaak RG, Hoadley KA, Purdom E, Wang V, Qi Y, Wilkerson MD, et al Integrated genomic analysis identifies clinically relevant subtypes of glioblastoma characterized by abnormalities in PDGFRA, IDH1, EGFR, and NF1. Cancer Cell. 2010;17(1):98–110. 10.1016/j.ccr.2009.12.020 20129251 PMC2818769

[5] Sturm D, Witt H, Hovestadt V, Khuong-Quang DA, Jones DT, Konermann C, et al Hotspot mutations in H3F3A and IDH1 define distinct epigenetic and biological subgroups of glioblastoma. Cancer Cell. 2012;22(4):425–37. Epub 2012/10/20. 10.1016/j.ccr.2012.08.024 . 23079654

[6] Moeckel S, Meyer K, Leukel P, Heudorfer F, Seliger C, Stangl C, et al Response-predictive gene expression profiling of glioma progenitor cells in vitro. PLoS One. 2014;9(9):e108632 10.1371/journal.pone.0108632 25268354 PMC4182559

[7] Neyns B, Sadones J, Chaskis C, Dujardin M, Everaert H, Lv S, et al Phase II study of sunitinib malate in patients with recurrent high-grade glioma. Journal of neuro-oncology. 2011;103(3):491–501. Epub 2010/09/28. 10.1007/s11060-010-0402-7 . 20872043

[8] Pan E, Yu D, Yue B, Potthast L, Chowdhary S, Smith P, et al A prospective phase II single-institution trial of sunitinib for recurrent malignant glioma. Journal of neuro-oncology. 2012;110(1):111–8. Epub 2012/07/27. 10.1007/s11060-012-0943-z . 22832897 PMC5735835

[9] Reardon DA, Vredenburgh JJ, Coan A, Desjardins A, Peters KB, Gururangan S, et al Phase I study of sunitinib and irinotecan for patients with recurrent malignant glioma. Journal of neuro-oncology. 2011;105(3):621–7. Epub 2011/07/12. 10.1007/s11060-011-0631-4 . 21744079 PMC3748953

[10] Kreisl TN, Smith P, Sul J, Salgado C, Iwamoto FM, Shih JH, et al Continuous daily sunitinib for recurrent glioblastoma. J Neurooncol. 2013;111(1):41–8. 10.1007/s11060-012-0988-z . 23086433

[11] Hutterer M, Nowosielski M, Haybaeck J, Embacher S, Stockhammer F, Gotwald T, et al A single-arm phase II Austrian/German multicenter trial on continuous daily sunitinib in primary glioblastoma at first recurrence (SURGE 01–07). Neuro Oncol. 2014;16(1):92–102. 10.1093/neuonc/not161 24311637 PMC3870838

[12] Tibshirani RJ, I.; Hastie T.; Efron B. Least angle regression. Annals of Statistics. 2004;(32):407–99.

[13] Pollard SM, Yoshikawa K, Clarke ID, Danovi D, Stricker S, Russell R, et al Glioma stem cell lines expanded in adherent culture have tumor-specific phenotypes and are suitable for chemical and genetic screens. Cell Stem Cell. 2009;4(6):568–80. Epub 2009/06/06. S1934-5909(09)00149-0 [pii] 10.1016/j.stem.2009.03.014 19497285

