# Supplementary figures and images for: Validation Study: Response-Predictive Gene Expression Profiling of Glioma Progenitor Cells In Vitro
Source: PLoS One. 2016 Mar 15;11(3):e0151312. doi: 10.1371/journal.pone.0151312 (PMC4792439; doi:10.1371/journal.pone.0151312)

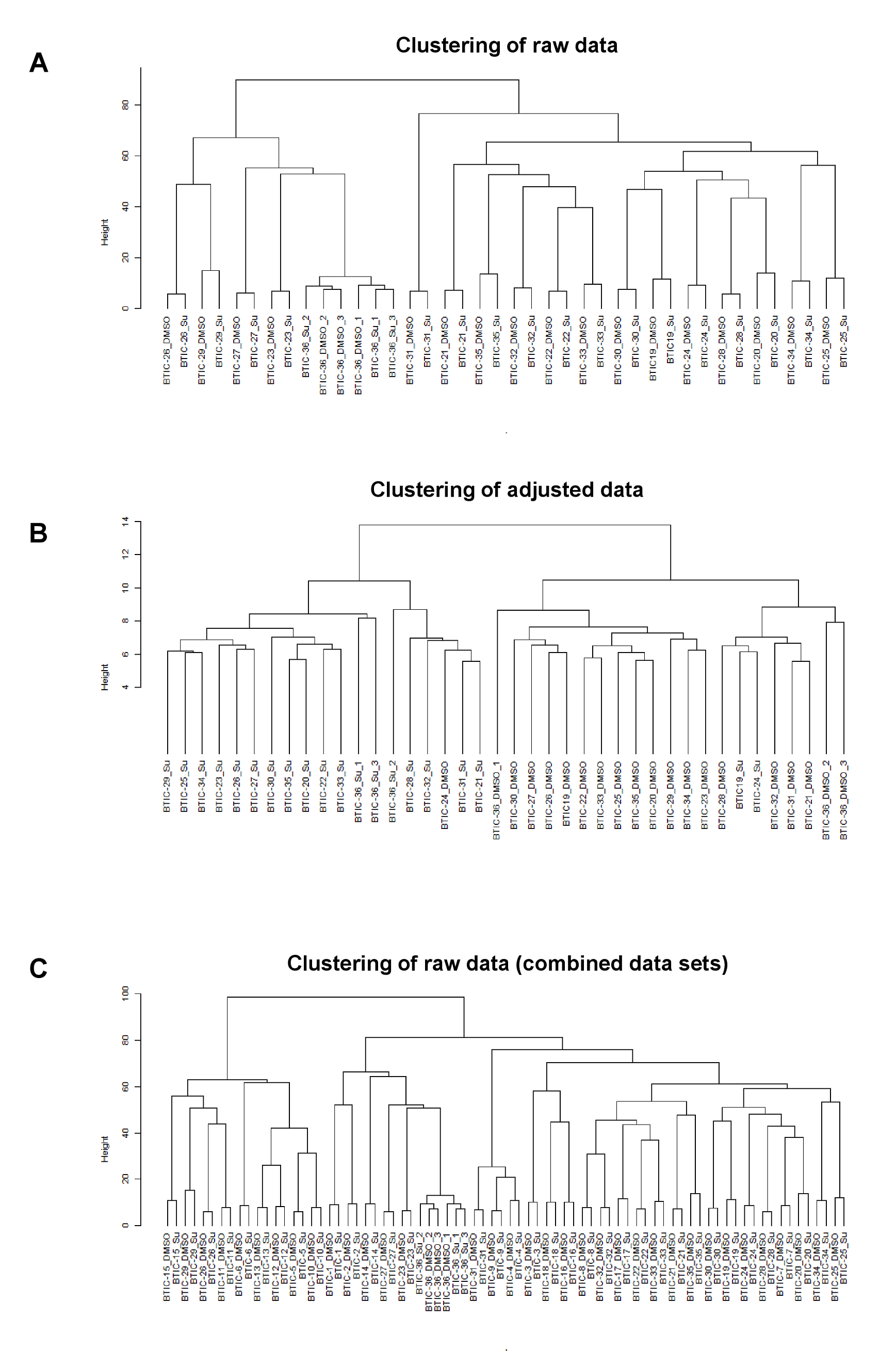

Supplement: S1 Fig — (A) The 500 most variable genes of the construction data set were hierarchically clustered according to Euclidean distances showing that treatment and control (DMSO) samples cluster within the corresponding BTIC line. (B) After compensation for inter-tumoral variability using the batch effect correction algorithm Combat, samples clustered mainly within treatment specific groups. (C) The hierarchical cluster of the 500 most variable genes of the combined dataset shows no separation of construction and validation samples. (TIFF) [file pone.0151312.s001.tiff]

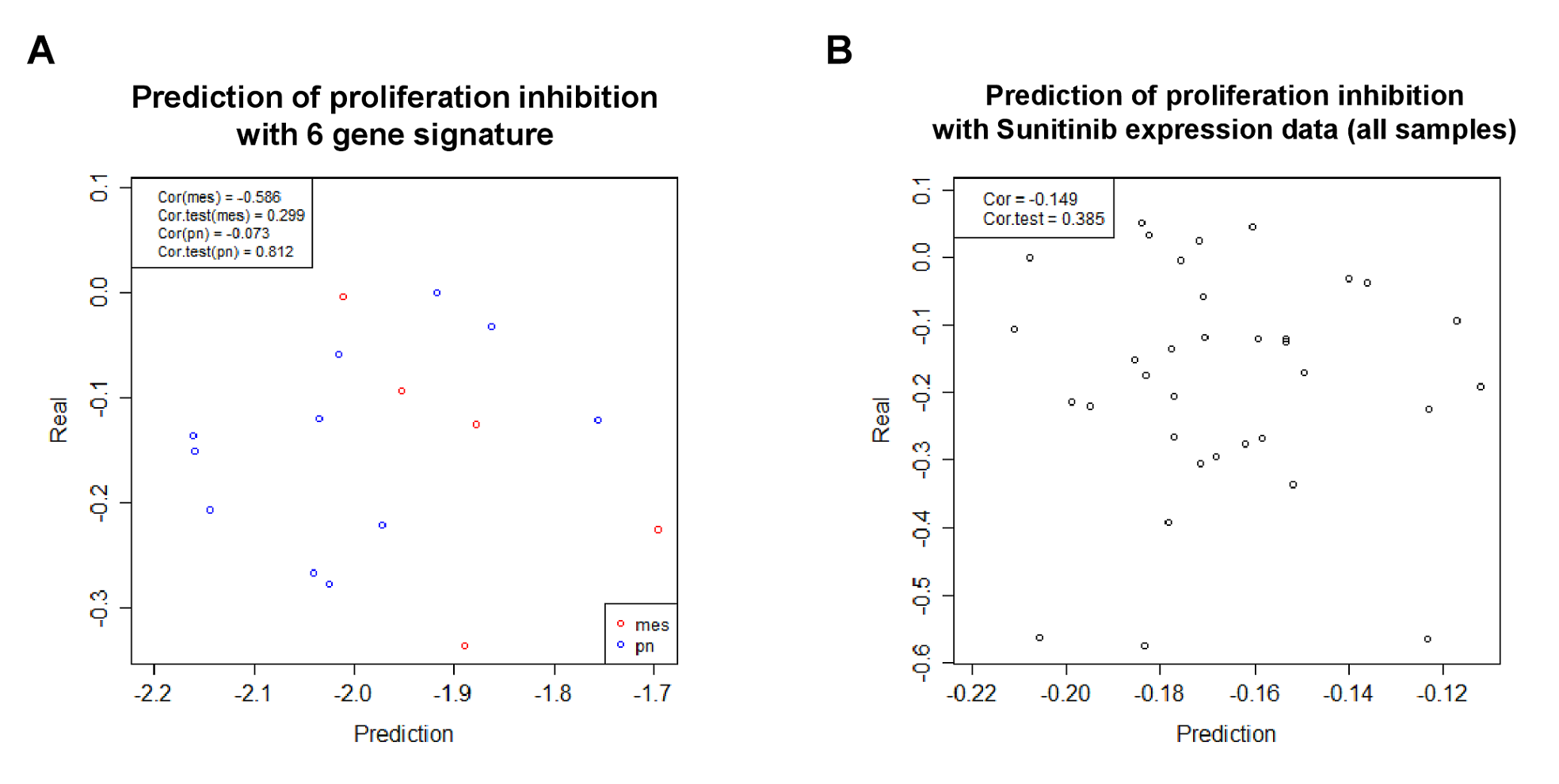

Supplement: S2 Fig — (A) Proliferation inhibition after 96 hours of treatment was predicted by calculating the sum of weighted expression of 6 signature genes (CLK4, BCLAF1, LOC100130581, ACTG, VAV3, DPF3). Predicted proliferation inhibition was plotted against the average relative proliferation inhibition (see Fig 2C). Data points corresponding to proneural BTICs were labeled blue and mesenchymal BTIC were labeled red, respectively. The Pearson’s correlation coefficients were calculated for each subclass separately and are shown in the upper-left plot legend. (B) Predicted proliferation inhibition was plotted against measured proliferation rates after treatment for all 36 BTIC cultures after running model selection with 36 samples as a training set. (TIFF) [file pone.0151312.s002.tiff]
